# GIANT PEDUNCULATED SEBORRHEIC KERATOSIS OF PENIS

**DOI:** 10.4103/0019-5154.39743

**Published:** 2008

**Authors:** Jagdeep S Thakur, Anamika Thakur, C G S Chauhan, Vijay K Diwana, D C Chauhan

**Affiliations:** *From Department of Plastic Surgery*; 1 *From Department of Pharmacology, IG Medical College, Shimla - 171 001, HP, India*

**Keywords:** *Penis*, *seborrheic keratosis*, *warts*

## Abstract

Seborrheic keratosis of the penis is a rare entity. It has been mistaken as genital warts and differentiation is only made on histopathology. We are reporting a case presenting as multiple giant polypoidal lesions on the penile skin for the last 20 years. Seborrheic keratosis should be considered in the differential diagnosis of pedunculated lesions of the penis. The histopathology after shave excision will be diagnostic.

## Introduction

The genital organs of the human body are prone to a variety of infective diseases which include bacterial, viral, fungal, protozoal. The infective rate of these diseases increase many folds depending on the sexual activity and immune status of the individual. The commonest viral disease in genital organs is condyloma acuminata caused by human papilloma virus (HPV). This virus involves the skin as well as mucosa of the genital organs. There other various non-infectious lesion of the genital organs. Seborrheic keratosis is one of rare non-infectious benign disease of the penis. We are reporting a rare and unusual case of seborrheic keratosis of the penis which can cause a diagnostic dilemma.

## Case Report

A 50-year-old male presented with large multiple polypoidal growths on the penis and groin region for last 20 years. Twenty years back he had progressive itching on the tip of the penis and within few days he developed small skin-colored lesions with clear fluid oozing from them on scratching. He consulted a dermatologist, who prescribed topical ointment and oral medicines. The patient got relief with these medicines and remained asymptomatic for five years. Then again similar lesions erupted on the prepuce first and subsequently over the whole length of the penis. These lesions were progressively enlarging, became hard, not associated with pain but were very itchy. For the last five months, the scrotum and groin region had also been affected. Patient was able to have sexual intercourse during the initial four years. The wife didn't complain of any such lesion on her sexual organs and was found normal on examination.

**Fig. 1:**
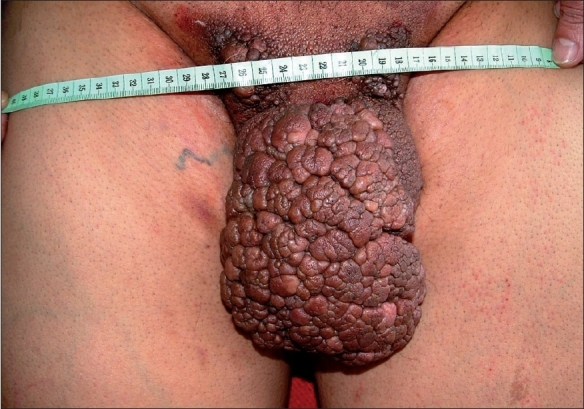
Giant penduculated lesion on the penis and groin area

On examination of the patient (Fig. 1), there were multiple polypoidal lesions on the skin of the penis, groin with average size of 2 cm. The intervening skin was normal and patient was able to urinate through slit-like opening. The scrotum skin was also involved but the lesions were not polypoidal.

**Fig. 2:**
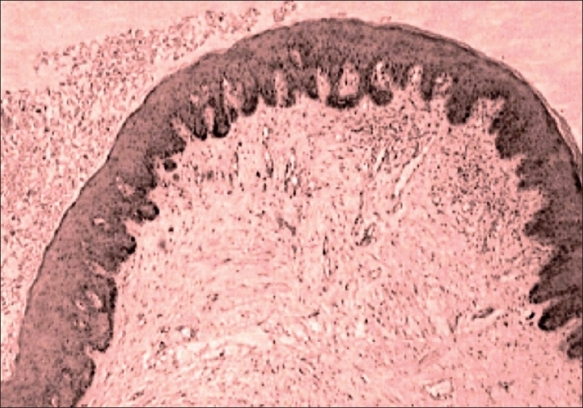
Photomicrograph showing finding consistent with seborrhiec keratosis (H&E, ×10)

**Fig. 3:**
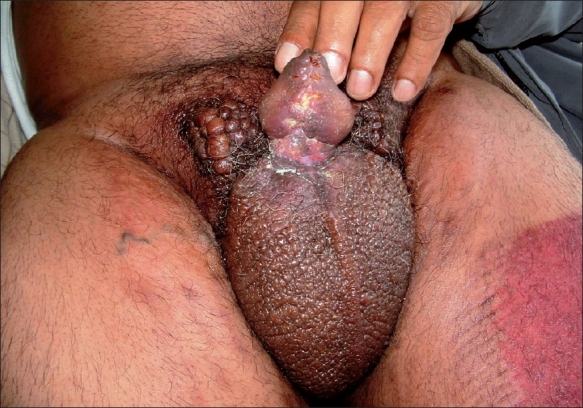
Photograph after a month of surgery, well-taken skin graft, healed donor site and residual lesion in groin area

The initial diagnosis of condyloma acuminata, acrochordons (Skin tags) and Bowen disease was kept and patient was investigated. The complete hemogram, liver, renal functions and immune status were found to be normal. The patient was taken for shave excision of the lesion under general anesthesia. The whole skin with lesion was excised up to the base of the penis. The thickness of the skin, excluding the lesion was about 2 cm. The mucosa of the penis was normal. The raw area was grafted with split-thickness skin graft. The histopathological examination of the tissue was found to be seborrheic keratosis (Fig. 2). On follow-up patient recovered well (Fig. 3).

## Discussion

We did a Pubmed Medline search with key words “seborrheic keratosis, penis, warts” and found only two reports of seborrheic keratosis in the penis.^1^,^2^ Stern *et al.*^3^ reviewed 527 lesions of seborrheic keratosis and found genital involvement in 2% but the gender has not been specified in the study. Seborrheic keratosis^4^,^5^ occurs after the third decade and affects only hair-bearing skin, invariably sparing the mucosal surfaces, the palms and the soles. They vary in color from tan to black. They can manifest as macules, papules, plaques or polypoidal lesions depending on the stage of development. Basal cell carcinoma, condyloma acuminata, melanoma are the most common misdiagnoses of seborrheic keratosis. Stern *et al.*^3^ reviewed 527 lesions and reported 49% accuracy by the clinician in diagnosing seborrheic keratosis.

There are reports^6^,^7^ of HPV in seborrheic keratosis-like lesions but it has also been reported that these lesions with HPV are actually condyloma acuminata.^8^

The treatment of seborrheic keratosis consists of shave excision, curettage, cryotherapy by liquid nitrogen, trichloroacetic acid application, electro dissection and carbon dioxide laser ablation.^4^,^9^

In our case report the seborrheic keratosis had a long duration of 20 years which was similar to the earlier report by Freidman *et al.*^2^ The patient had no mucosal involvement. The wife had no such lesions hence excluding genital warts as these are contagious. Acrochordons (skin tags) are found in about 25% of adults. They appear in the second decade with progressive increased frequency up to the fifth decade and are found mostly in the neck or axilla but may occur in the groin or limbs as isolated large polypoidal lesions. They occur as multiple dark or skin-colored, filiform or smooth-surfaced papules, approximately 2-3 mm in diameter. The stalk of skin tags is fibrous and consists of loose connective tissue with dilated capillaries. The Bowen disease is a premalignant condition of the skin (also known as squamous cell carcinoma *in situ*). It is mostly found in the lower limbs of females and scalp or ears of males. Characteristically it is well defined, slightly elevated, red, scaly plaque with fissured surface. The progression of this disease is slow by lateral extension invading the dermis and creating ulcer.^4^,^5^,^10^

## Conclusion

The penis is rarely affected by seborrheic keratosis. This case report presented a rare form of seborrheic keratosis which affected the penis. This rare condition should be considered in the differential diagnosis for the lesions of the penis and histopathology after shave excision will help in the diagnosis.
